# Personal and Societal Recovery in Depression—A Qualitative Study

**DOI:** 10.1111/inm.70247

**Published:** 2026-03-19

**Authors:** Annelot van Geffen, Dorien Smit, Janna N. Vrijsen, Jan Spijker

**Affiliations:** ^1^ Pro Persona Mental Health Care, Depression Expertise Center Nijmegen the Netherlands; ^2^ Behavioural Science Institute Radboud University Nijmegen the Netherlands; ^3^ Research Group in Social Psychiatry and Mental Health, HAN University of Applied Sciences Nijmegen the Netherlands; ^4^ Department of Psychiatry, Donders Institute for Brain, Cognition and Behaviour Radboud University Medical Center Nijmegen the Netherlands

**Keywords:** depressive disorder, mental health recovery, psychosocial rehabilitation, qualitative research, social participation

## Abstract

There is growing attention for personal and societal recovery in mental health care. Existing research in this area is primarily focused on populations with Severe Mental Illness (SMI), while the development of personal and societal recovery in depression remains largely unstudied. This study explored the development of personal and social recovery, barriers and facilitators, and ways in which the recovery process can be supported for people with depression. Fourteen semi‐structured interviews were conducted with individuals who experienced at least two episodes of depression and were in remission at the time of the interview (except for two deviant cases). Data were analysed using a constructivist grounded theory (CGT) informed approach combined with elements of reflexive thematic analysis (RTA). Purposeful sampling was applied to gather a heterogenous sample and data collection continued until theoretical saturation was reached. COREQ guidelines were followed. Participants described personal recovery as an intrapersonal, retrospective process of self‐discovery. Societal recovery was described as an interpersonal, prospectively oriented process centred around developing belonging. These processes are bi‐directional, as societal recovery created further opportunities for personal recovery. Participants described a preliminary step of ‘societal participation’ involving low demanding tasks to rebuild functional capacity and self‐esteem. During personal recovery, participants need empathetic support which transitions to pragmatic support as individuals progress towards societal recovery. Our findings underscore the interrelatedness of personal recovery and societal recovery. Self‐insight, developed through personal recovery, is applied during societal recovery. This occurs through the alignment of activities with personal values and meaning. Belonging in society through meaningful participation is what drives people towards societal recovery. Societal recovery often develops through an important step of societal participation.

**Abbreviations**
CGT: constructivist grounded theory
GT: grounded theory
MINI interview: Mini International Neuropsychiatric Interview for DSM‐IV‐TR
OPH: occupational perspective of health
RTA: reflexive thematic analysis
SMI: serious mental illness
TA: thematic analysis

## 1 Introduction

Depression is highly prevalent (Dattani et al. 2023) and comes with a huge personal and societal burden (Greer et al. 2010). Traditionally, treatment of depression prioritises clinical recovery: the reduction of clinical symptoms and related behavioural and cognitive problems (Whitley and Drake 2010). However, a recent meta‐analysis (Cuijpers et al. 2024) demonstrated that clinical recovery (50% or more symptom reduction) is often not achievable, urging the importance of a broader perspective on recovery in mental health. The recovery approach, originating from the user perspective as a movement (Slade et al. 2008), posits recovery as a multidimensional, personal process by including personal and societal recovery dimensions in addition to clinical recovery. This recovery approach has gained significant momentum in recent years, both in policy and in clinical practice (Ahmed et al. 2012). Among mental health professionals, however, a systematic review showed that there is still a lack of clarity about the concept of recovery and the implications for clinical practice (Gyamfi et al. 2020). As the recovery approach is becoming increasingly integrated in clinical practice, understanding how this process develops and can be supported for people with depression is essential.

## 2 Background

Personal recovery is defined as a process of ‘changing one's attitudes, values, feelings, goals, skills, and/or roles. It is a way of living a satisfying, hopeful and contributing lie even with the limitations caused by illness’ (Anthony 1993, 8). Important elements of the personal recovery process were later distinguished through the acronym CHIME: Connection, Hope, Identity, Meaning and Empowerment (Leamy et al. 2011). The concept of recovery is mostly studied in populations with Serious Mental Illness (SMI) such as patients with psychotic disorder (Castelein et al. 2021; Leendertse et al. 2021; Mitsunaga‐Ohmuro and Ohmuro 2021) and bipolar disorder (Kraiss et al. 2019, 2021). And while recovery is generally considered a personal journey independent of the specific mental health condition, studies have found specific elements attributed to the personal recovery process in depression. For example, quantitative evidence suggests that depressive symptoms in particular impact personal recovery (Van Eck, Burger, Schenkelaars, et al. 2018; Van Eck, Burger, Vellinga, et al. 2018). Qualitative studies on the recovery process in depression found that authenticity (Ridge and Ziebland 2006), social and existential aspects, such as belonging to a (social) group (Damsgaard et al. 2021; Richardson and Barkham 2020) and the development of experiential knowledge by introspection and self‐management (Smit et al. 2020) are important aspects of personal recovery in depression.

Depression often comes with ‘profound ruptures to what the person was living for—their dreams for work, relationships, and a meaningful life’ (Desai et al. 2019, 275). Societal recovery aims to restore these ruptures by being an active participant in society through (volunteer) work, study or other activities and social roles (Whitley and Drake 2010). Studies have shown a bi‐directional relationship between societal participation and mental wellbeing (Ding et al. 2015; Nagata et al. 2021), showing the importance of societal participation. In addition, understanding how to support societal recovery in depression could potentially help lower costs associated with absence due to depression, estimated at 1.8 billion euro yearly (Ministerie van VWS and RIVM 2017) in The Netherlands. As research in depressive populations is limited, we explored what personal and societal recovery entail for people who experience(d) depression, how it develops, barriers and facilitators, and how this process can be supported by mental health professionals, the social context and peer support.

## 3 Methods

### 3.1 Aim of the Current Study

This qualitative study explored how personal and societal recovery develops for people with depression. The research questions guiding our inquiry were:

1. What does personal and societal recovery entail for people who experience(d) depression?
2. How are the processes of personal and societal recovery related?
3. What barriers and facilitators can impact the process of personal and societal recovery?
4. How can the processes of personal and societal recovery be supported by mental health professionals, the social context and peer support?

### 3.2 Design

Fourteen semi‐structured interviews with individuals who experienced depression were conducted following a self‐developed interview guide (see Appendix S1). A constructivist grounded theory (Charmaz 2014) informed approach was taken that can be utilised to assess patients’ lived experience and inform clinical practice (King et al. 2025). This was combined with elements of a reflexive thematic analysis framework (Braun and Clarke 2006, 2019). As proposed by Braun and Clarke (Braun and Clarke 2006), TA can be used within other analytic traditions, such as GT (Chapman et al. 2015). This hybrid approach aided in developing a conceptual model, rather than merely describing the data through themes. It allowed us to begin from the existing conceptual themes of ‘personal and societal recovery’, which we treated as ‘sensitising concepts’ (Boeije 2011; Zaidi 2022). With this approach, the general meaning of these concepts was used as a framework while remaining open to inductively exploring their meaning for people with lived experience of depression. Our interview guide contained open questions on these sensitising concepts (see Appendix S1). Data collection and analysis occurred in a cyclical manner, and elements of theoretical sampling were applied to recruit participants with additional or missing perspectives. Recommendations from the COREQ checklist for qualitative research were followed (Tong et al. 2007), see Appendix S2.

### 3.3 Participants

Inclusion criteria were: two or more previous episodes of depression, willingness to reflect on own recovery journey, 16 years or older, able to sign informed consent form. Exclusion criteria were: a current depressive episode (to minimise burdening participants), acute suicide risk, psychotic disorder, bipolar disorder, current substance dependence, or inadequate proficiency in Dutch. Participants were included based on various demographic (gender, age, ethnicity) and clinical characteristics (number of episodes, current treatment, recovery) to obtain a heterogeneous sample. These characteristics were assessed through an online questionnaire. Further eligibility was assessed during a telephone screening through administration of the MINI (Mini‐International Neuropsychiatric Interview, Dutch Translation) (Vliet and de Beurs 2007). Two deviant cases were included that differed from in‐ and exclusion criteria to enrich our data and enhance credibility: one participant with only one episode of depression and one participant with a current depressive episode.

### 3.4 Procedure

Participants were recruited through two associations for depression and/or mental health in the Netherlands: The Depression Association (Depressie Vereniging) as a patient association and Ixta Noa as a recovery‐oriented initiative. The study was advertised in the newsletter of both organisations and posted on their social media platforms. In addition, the study was advertised on the online peer support community Depression Connect (Smit et al. 2021). Participants were asked to contact the researcher by e‐mail when they were interested in participating in the study. Participants were then asked to fill out a short online questionnaire with questions on demographic and clinical variables. Additionally, participants were asked to give consent for their responses to be shared with the researcher and to allow the researcher to contact them for participation in the study. When participants met inclusion criteria, an appointment was made for the individual semi‐structured in‐depth interview. The interviews were held in Dutch, online or at a location of choice of the participants, and could last up to a maximum of 90 min. The interviewer would start with a general description of the aim of the study, the background of the interviewer, and the process of the interview (see Appendix S1). Participants were asked consent for the interview to be recorded on a hand‐held audio recorder in addition to the signed informed consent. One pilot interview was held (which was excluded from data analysis) to test the length and duration of the interview. The interviews were transcribed verbatim, and the transcripts were anonymised. The first author conducted all 14 interviews and did not have a relationship with the participants prior to the time of data collection. In addition, participants were invited to optionally take part in an individual member‐check, where preliminary results would be presented for feedback to enhance trustworthiness.

### 3.5 Participant Characteristics

In Table 1, demographic and clinical variables of the sample can be found. We interviewed 10 female participants and 4 male participants, who were between 24 and 58 years old. Half of the participants experienced 3–5 depressive episodes. All participants had been in treatment for their depressive symptoms in the past, and some still received (maintenance or relapse prevention) treatment at the time of interview.

**TABLE 1:** Demographic and clinical characteristics of participants (*n* = 14).

Gender	Male	4
	Female	10
Age	Mean	43.6
	Range	25–58
Ethnicity	Dutch	12
	Moroccan	1
	Chinese	1
Education	Secondary education	1
	Secondary Vocational Education and Training	4
	Higher education (research‐oriented and profession‐oriented)	9
Treatment history	Treatment history (therapy)^a^	14
	Current pharmacotherapy for depression	8^b^
	Current therapy	6^b^
Number of previous depressive episodes	One episode (negative case)	1
	Two episodes	3
	3–5 episodes	7
	> 5 episodes	1
	Chronic course only	2
	Chronic course in addition to depressive episodes	8
Age at onset, years	12–18	7
	19–25	3
	26–32	1
	33–45	3
	≥ 46	0
Occupational status	Paid employment	6
	Volunteer work	2
	Paid + volunteer work	2
	Study (including side jobs + volunteer work)	2
	None	2
Living situation	Alone	5
	With partner	9

^a^ Treatment includes psychotherapy and pharmacotherapy.

^b^ Including overlap. Participants currently in treatment were in remission and received ongoing pharmacological and/or psychological care as part of maintenance or relapse‐prevention regimens, instead of receiving active treatment for depressive symptoms.

### 3.6 Data Analysis

Interview transcripts were analysed using Atlas.ti version 22.2.2 for Windows. The coding process involved assigning codes to the data through a process of initial, focused and theoretical coding (Boeije 2011; Charmaz 2014; King et al. 2025). In the initial coding stage, each relevant transcript segment was summarised in a broad descriptive code capturing its meaning. Each interview was independently coded by the first author AG, a female junior researcher (MSc) with theoretical understanding of the topic and a research assistant (clinical psychology intern at Pro Persona) with limited knowledge on the topic. Each interview would be discussed line‐by‐line and all given codes would be reviewed until reaching consensus about its interpretation. After coding three to four transcripts, initial codes were reflected upon through memos and to enhance reflexivity, discussed with the second coder. Initial codes were then organised into broader categories and subcodes, forming a hierarchical thematic structure. The organisation of codes into hierarchical themes was conducted as part of our hybrid approach (CGT combined with RTA) and was used to structure and present the findings. Themes were iteratively revised through reflexive discussions with the two research teams. The first team consisted of experienced researchers with expertise in mental health care, including a senior psychiatrist. The second team consisted of members of the project team which involved multiple experts by experience from recovery initiative Ixta Noa and the Depression Association. In this way, we aimed to enhance the trustworthiness of the study by staying close to the experience of people with depression themselves. Intermittent results determined the research direction through comparison with earlier results. This process occurred iteratively until theoretical saturation was reached (Boeije 2011). Through theoretical coding, relationships between themes were assessed and were integrated into a conceptual model describing participants' recovery processes. All 14 participants were also invited to participate in member‐checks, of which seven were held, consisting of online individual interviews, where initial findings were presented to participants, soliciting their feedback on the findings and bring further nuances (Boeije 2011). No participants dropped out of the study. Results are synthesised and presented through a narrative storyline (Birks et al. 2009).

## 4 Results

**FIGURE 1:**
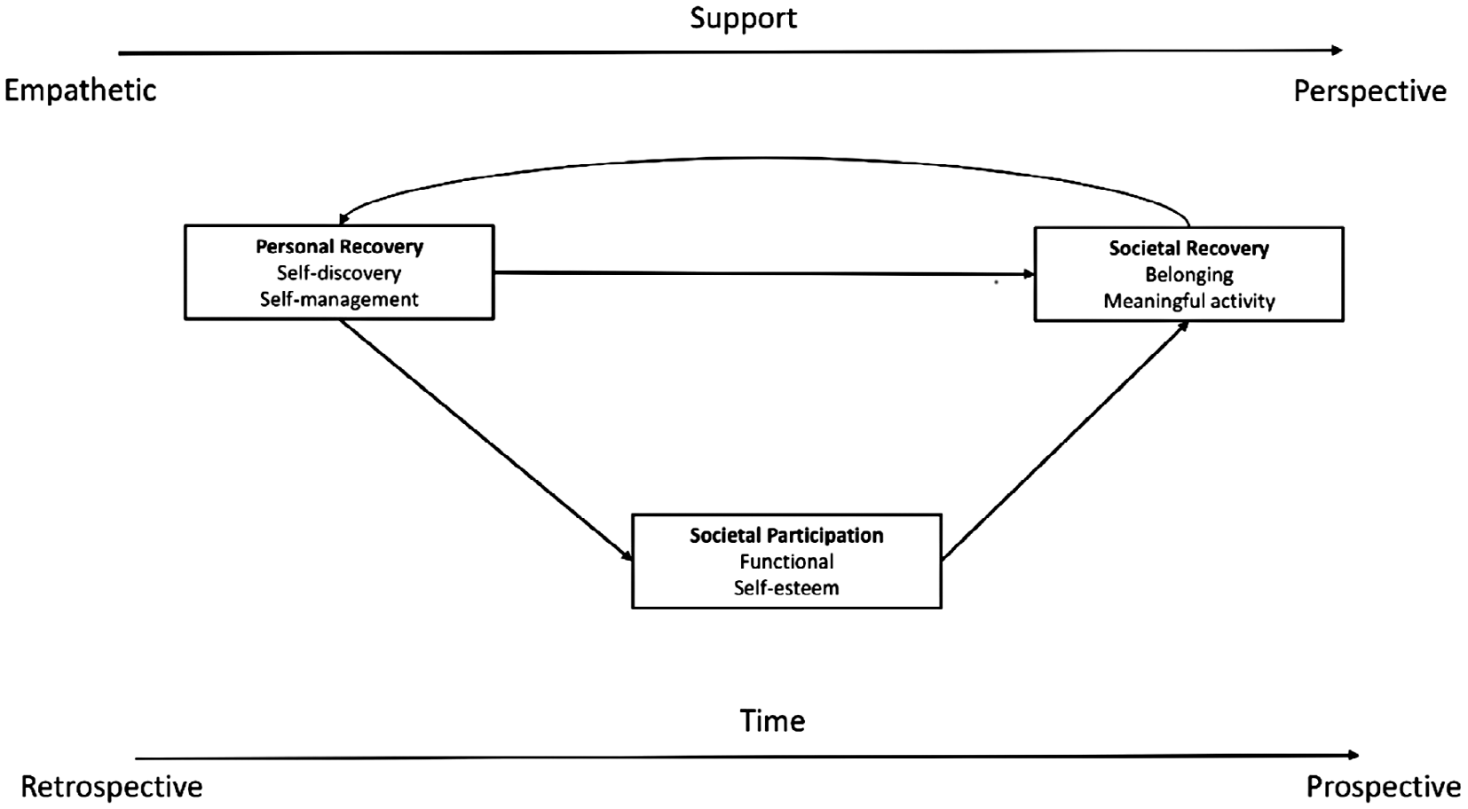
Visualisation of the conceptual model for the development of personal and societal recovery in depression.

An overarching conceptual model describing the processes of personal and societal recovery in depression is visualised in Figure 1. Themes and core categories that became apparent through analysis are presented in italics in the text.

### 4.1 The Recovery Process: A Complex and Personal Journey

Participants described that their recovery journey was characterised by finding a way to *deal with persisting symptoms* and *living a meaningful life* despite it. Participants would describe the recovery process as a *personal* and *complex* journey going from merely ‘surviving’ to ‘thriving’ and ‘being able to *live a normal life*’.

Female, 46 years old: ‘And I think that's… recovery. Not that you'll never feel down or have a bad day again, but that you do know how to deal with it and that you also have the confidence that it will pass again… yes, I think that's what recovery is.’

### 4.2 Personal Recovery: Developing Self‐Insight and Deploying Self‐Management Strategies

Personal recovery was experienced as an *intrapersonal* process of *self‐discovery*, developing *self‐insight* and *self‐management*. Developing self‐insight was a retrospective process, by understanding where and how (*negative) patterns* were formed in the past. Through this process participants developed *an improved relationship with themselves*. Participants described it as a way of getting to know themselves in their ‘*core*’ by gaining a deeper understanding into one's *identity*, becoming in touch with their *desires and needs*, letting go of their identity as being ‘a depressed person’ and being *empowered* in their discovered identity by having a more positive *self‐image* and gaining *self‐esteem*.

Female, 26 years old: ‘You're going to believe more and more in that depression and everything and in that sense, recovery is, I guess, more and more back to you as a person. Instead of you as a depressed person…’

Participants described a sense of having *lost oneself* at the start of their depression, through *sacrificing own needs* to accommodate others. This resulted in a feeling of having to wear a ‘*mask*’ all the time, which caused a sense of *isolation* from others. Because of this *self‐alienation*, the process of *self‐insight* and *discovery* held great importance and allowed participants to start *prioritising themselves and their needs*, which was important to their recovery.

Female, 30 years old: ‘It's a bit like they say on an airplane, right? You have to put your own oxygen mask on first before helping your child. I think that's a real lesson… I always wanted to take care of others, but I never learned to take care of myself. And in the end, I lost myself. It's like I, sort of, ran away from myself and, yeah, completely lost… well, lost myself, you could say.’

However, this gained *self‐insight* had to also be put into practice by making *conscious choices* that are in alignment with their insights. Applying the insights through deliberate actions is an important next step and unfolds through the deployment of *self‐management strategies*. Self‐management strategies were described on 4 levels: *mental* (acceptance), *physical (*being active), *emotional* (allowing feelings) and *environmenta*l (letting go of judgement from others).

Female, 30 years old: ‘Yes, that I have confidence in it, that I have now developed a reasonable number of skills for this [avoiding a relapse]. Yes, but that's why, that I have the skills to, at least, do something with this’.

### 4.3 Societal Recovery: Fostering Belonging Through Meaningful Participation

Societal recovery was described as a process of *(re)joining* society and actively engaging as a participant within it. Societal recovery was described as an *interpersonal* and *prospective* process. In the process of societal recovery, participants were able to look prospectively at the future and regain *hope* for a more positive future in which they felt part of society again. An important aspect that participants noted as central to societal recovery is *belonging*. While *financial benefits* and *being independent* were motivating for two participants, the main motivation for societal recovery was to foster a sense of *belonging* through *participation* and being able to *contribute to society* in a *meaningful way*.

Female, 25 years old: ‘Um, I think on all fronts, participation. Feeling like you're participating, um and whether that's with work or volunteering or studying, I think, it doesn't even matter so much. But the feeling that you're part of society and that you contribute something to it and yes, I felt so distant from everything and everyone at one point. That yes, then you, you don't take part in society anymore or something and also feeling the connection with people, I think that's also part of it, that you feel connected to people and therefore also maybe feel a reason to want to live or something, but especially, yes, participating. That's so important.’

Societal recovery was thus characterised more in terms of experiences, such as a sense of *meaning* and *belonging*, rather than by traditional markers of achievement, such as paid employment. For two participants with a non‐Dutch ethnicity, belonging also entailed finding your place in a *different culture*.

### 4.4 Interrelated Processes of Personal and Societal Recovery

The process of personal and societal recovery appeared to be interrelated, creating a bi‐directional dynamic. Personal recovery is essential to societal recovery as it involves developing *self‐insight* into what is meaningful to the individual through *self‐discovery*. Societal recovery provided participants with opportunities, such as a (volunteer) job, study or other activities, that *aligned* and were *fitting* with their core *identity and values*, as uncovered in the personal recovery process. Societal recovery became a way to engage with society in accordance with one's authentic self, which would foster a sense of *belonging*. For some, this would directly counter the *isolation* and *alienation* experienced during depressive episodes. Societal recovery would also instigate a new phase of personal recovery by providing opportunities for ongoing personal growth, creating a dynamic and two‐way interaction between personal and societal recovery.

Male, 57 years old: ‘Because recovery also has a lot to do with finding the right place, finding or doing something that falls into that congruence [with your identity], that aligns with your core. Because otherwise, you're talking about surviving and not about thriving’.

### 4.5 Societal Participation: A Preliminary Step Towards Societal Recovery

Participants frequently described an intermediate phase of *societal participation* as a preliminary step towards societal recovery. In this transitional phase, termed ‘*societal participation*’, participants engaged in minimally demanding, low‐pressure flexible activities. This phase emphasised active involvement without *pressure*, rather than the inherent *congruence* of tasks and settings with *individual values*. The primary goal during this period was to strengthen participants' functional capacities, as many experienced *lingering (cognitive) symptoms* and *limited taxability* due to the depression. During this phase, participants would take *small incremental steps* to gradually increase their activity level, capacity and consequently, build a sense of *self‐confidence*. However, as participants progressed, they became dissatisfied with these activities as they lacked *growth opportunities* and *alignment* with personal values. This marked a transition towards societal recovery, where participants made *conscious choices* regarding their social roles and activities that were more in *congruence* with their *identity and values*.

Female, 27 years old: ‘That [volunteer work] helped me back then, I was… it was also a part of my daily routine, so I went there [place of participation] and then I would do that. So, it was very good for me, but I also noticed a bit of, yeah, lack of satisfaction.’

### 4.6 Barriers and Facilitators in Societal Recovery

Participants described several barriers and facilitators to societal recovery. As mentioned, taking *incremental steps* was facilitating, allowing participants to regain *trust* and *confidence* in themselves. It was important to participants to be *autonomous* and not *pressured*, to reintegrate at *their own pace* and have enough *rest*. *Activation and structuring* techniques such as establishing clear objectives, maintaining *scheduled commitments*, and adhering to a daily *routine* proved beneficial. A clear and concrete *future perspective* was also helpful for participants. The balance between getting enough *outside of one*'*s comfort zone* and being challenged while *not being overwhelmed* at the same time was important.

However, several barriers were found to form possible obstacles to progress in societal recovery. *Fear of relapses* and *failure* and the *inability to restore to previous levels* of functioning were described as hindering. *Internal pressures*, such as perfectionistic tendencies along with *external pressures* from *social security institutions* or *employers*, presented significant challenges. *Financial challenges* could also pose a hindrance to recovery by inducing stress and instability. *Lingering symptoms*, such as diminished concentration, further complicated the trajectory towards societal recovery. In addition to these general factors that would facilitate or hinder progress in recovery, professional, social and peer support were specifically studied.

### 4.7 Professional Mental Health Care in Personal and Societal Recovery

During the phase of personal recovery, professional support was noted by participants as helpful. Therapy could facilitate the process of *self‐discovery* by understanding one's (negative) *patterns*, *coping* and *thought patterns*. Offering *hope* that change is possible was also noted as important.

Female, 34 years old: ‘That someone gave perspective. That was the psychia… that was the psychiatrist, who then says: “hey, I think there are ways for… for things to get better.”

And that was also the moment that I felt something like hope for the first time.

And, like, maybe this is the moment to actually take a step forward.’

Participants described that professional support was less helpful in their recovery when experiencing not being *understood* or taken seriously by their therapist or *not having autonomy* in choices. Specifically, post‐treatment, participants noted that the transition to live on their own and having less daily routine was hard to navigate as this phase coincided with less frequent professional support opportunities. The transition towards independent living in society left a notable gap, leaving individuals to navigate this phase largely independently.

Male, 32 years old: ‘So if I just sat in the mental health facility 24 hours a day, 5 days a week, it was super easy, of course. Because I had to do groceries, I had to get up, I had to go to that therapy, and if you didn't come, they would come and get you, so to speak. So there's nothing easier than that. And when you're at home, suddenly you're responsible for all those things yourself and um yeah, that's just…Yeah, that's really really very… a whole different ball game of course.’

### 4.8 The Social Context in the Recovery Process

Participants described ways their social context could both facilitate their recovery process as well as hinder it. Facilitating factors regarding social support include *empathy* and *understanding*, *being seen and heard, being non‐judgmental, listening, receiving positive input, reflecting, connection and safety*. Barriers regarding the social environment include a sense of *not being understood, stigma and financial and institutional barriers*. With close contacts such as family members or a partner, a sense of *guilt* was also experienced as hindering, being *dependent* upon them (financially) and receiving *unsolicited advice. Employment* could both be a risk factor as well as a protective factor. Several participants noted that work was the factor that propelled burn‐out and depressive symptoms by *heavy work pressure*, using work as a *coping mechanism*, or because of working in *unfulfilling roles* that did not align with their *personal values*. On the other hand, several participants described that employment was their safe haven. It provided them with a sense of *identity, value and structure*. Multiple participants expressed feelings of *guilt* when being on sick leave, influenced by a deeply ingrained *work ethic* that emphasises the necessity of labour for financial remuneration.

Female, 44 years old: ‘Hard work, ensuring that you, that you are seen in that way. Yes, that was my coping.’

### 4.9 Peer Support: Empathetic and Learning Environment

Participants who used peer support opportunities such as the online peer support community Depression Connect or support groups described peer support as particularly helpful in offering an *empathetic environment* where *mutual understanding* and *acknowledgment* could offer relief from the sense of *isolation* many participants experienced. Exchanging experiences with peers was also found to stimulate *learning* from others in how to deal with depression.

### 4.10 Different Support Needs During Personal and Societal Recovery

However, member‐checks revealed that participants had different support needs at different phases of their recovery. While peer support was found to be specifically beneficial during the personal recovery phase by offering *empathetic understanding* and *acknowledgment*, this need seemed to decrease during societal recovery. During societal recovery, several participants described they initially joined a peer support group but discontinued their involvement, perceiving it as less beneficial or even harmful in their process towards societal recovery. Participants noted they perceived themselves to have surpassed the stage of seeking *empathy* and *recognition*. In this phase, they instead expressed a need for *pragmatic guidance* on societal reintegration and *future perspective* while also prioritising *active listening* and fostering *insight* through *self‐discovery*. Importantly, such support must be *tailored* to each individual, devoid of *rigid protocols*.

Female, 57 years old: ‘Maybe it also comes from the fact that you… have to be careful, I remember in the past, if I then… was in crisis, I don't remember where it [the peer support group] was from.. But that you drag each other down.’

## 5 Discussion

Our data suggest that personal recovery is an important precursor to societal recovery, and societal recovery in turn provides further opportunity for personal recovery. Personal recovery appeared to be an intrapersonal, retrospective process of developing self‐insight and self‐management strategies. Self‐insight allows societal recovery to develop by undertaking activities that align with personal values. Societal recovery is a prospective process of fostering a sense of belonging in society through meaningful activity. Societal recovery specifically develops through a transitional phase that we termed ‘societal participation’. Finally, the recovery process can be facilitated by different types of support. Empathetic support, through listening and being supportive and non‐judgmental, is specifically important during personal recovery. During societal recovery, more practical guidance and a realistic perspective are sought after.

### 5.1 Personal Recovery as a Transformation of ‘Self’

Earlier qualitative research supports our findings, suggesting that recovery is a complex and personal journey (Fernández et al. 2023; Richardson and Barkham 2020) involving self‐discovery (Fernández et al. 2023; Richardson and Barkham 2020) and self‐management strategies (Smit et al. 2020). A key finding from our study is the improved relationship that participants experienced towards themselves, progressing from feeling alienated from oneself towards feeling empowered to express their authentic selves. Earlier findings also suggest that authenticity (Ridge and Ziebland 2006) and the construction of ‘self’ (Richardson and Barkham 2020) are important. Fernández et al. (2023, 6) called this the ‘transformation of the experience of the depressed self’. Several studies (Kopala‐Sibley and Zuroff 2020; Luyten and Fonagy 2016) outlined theories to explain the relationship between the ‘self’ and depression. Across these theories, it is postulated that a ‘failure to separate one's sense of self from external standards as well as the consistent pursuit of extrinsic over intrinsic goals likely lead to feelings of low self‐worth, interpersonal loss and consequently, depression’ (Kopala‐Sibley and Zuroff 2020, 21). Our study supports the notion that reconnecting to oneself is essential in depression, as people with depression tend to experience a disconnection from self (Mancini et al. 2024) and others (Osler 2022). We add to the existing literature that this self‐discovery is an important aspect of personal recovery in depression. In addition, we found that the process of self‐discovery is an important precursor to societal recovery and participation.

### 5.2 Societal Recovery: Belonging Through Doing, Being and Becoming

Belonging through engaging in meaningful activities appeared central to societal recovery in our study. Several studies have shown that belonging plays a significant role in depression (Dutcher et al. 2022; Fisher et al. 2015; Parr et al. 2020; Steger and Kashdan 2009). Our findings add to the literature that in depression, developing a sense of belonging is an important aspect of societal recovery. This aligns with the Occupational Perspective of Health (OPH) (Hitch et al. 2014; Wilcock 1999) that describes this process as ‘Doing‐Being‐Becoming‐Belonging’. ‘Doing’ relates to our findings that participation is an essential human need. ‘Being’ refers to our findings that participation has to align with someone's identity and personal values, which offers opportunity for further growth, or ‘Becoming’. This in turn fosters a sense of connection with others through ‘Belonging’. In our study in a depressive population, belonging is both a motivation for people with depression to participate as well as an outcome.

### 5.3 Societal Recovery in the Context of the CHIME Framework

Predominantly used for personal recovery, our qualitative analysis shows that the principles of the CHIME framework (Leamy et al. 2011) are also applicable to societal recovery. Specifically, connectedness was described in terms of a sense of belonging within society and feeling connected to others. Hope for a better future was closely linked to possibilities for societal participation. Identity emerged as essential, as social roles and activities need to be congruent with one's sense of self. Meaning was often derived from participation in aligned activities, and empowerment was reflected in regaining control and agency as an active member of society. Our findings support the suitability of the CHIME framework in the context of societal recovery. Further research is needed to understand if societal recovery can be seen as a distinct concept from personal recovery.

### 5.4 Societal Participation as a Transitional Phase in Recovery

While societal recovery refers to participation in a way that aligns with one's values and identity, our findings suggest that participants often experience a transitional phase of ‘societal participation’. In this phase, activities are undertaken that allow participants to increase their taxability in small incremental steps and build a sense of self‐confidence and trust, before transitioning to societal recovery. This intermediate step relates closely to the concept of ‘functional recovery’ (Van Aken et al. 2023) in that it aims to strengthen cognitive functionality that was impaired due to depression. We add to the current literature by describing that societal participation, is not yet full recovery, but often an important intermediate step towards societal recovery. Welfare institutions and rehabilitation services, including return‐to‐work programmes, are often involved in the phase of societal recovery, and our findings have important implications for professionals in these fields. First, it is essential to recognise that a sense of belonging is a key motivation for individuals returning to work or other societal roles. Facilitating self‐discovery and encouraging activities aligned with an individual's values are crucial elements of this process. Additionally, incorporating an intermediate ‘societal participation’ phase with flexible, low‐demand tasks can help rebuild an individual's capacity and self‐confidence, tackling the fear of failure many experience. Taking these aspects into account when supporting people with depression with societal recovery can be beneficial.

### 5.5 Supporting the Recovery Process

We found that in the process of personal recovery, emphasis has been placed on empathetic (peer) support, transitioning towards more pragmatic support during societal recovery. Meta‐analytic findings (Smit et al. 2023) offer some evidence for this. Their study included 28 randomised controlled trials (RCTs) involving 4152 individuals and revealed that peer support demonstrates significant post‐test effect sizes for clinical and personal recovery, yet not for functional recovery, which in this study relates more closely to societal participation and recovery. Our study suggests that, while peer support initiatives are highly beneficial for personal recovery, they often lack the pragmatic and concrete support that would be especially valuable during societal recovery. This support should be tailored to the individual, being realistic and practical, while also prioritising active listening, offering perspective and facilitating self‐discovery. These aspects correspond largely to the working mechanisms of Recovery Oriented Practices laid out in a review by Winsper et al. (2020). Barriers within the social environment, including a sense of not being understood, stigma, and financial and institutional obstacles, have been identified as significant impediments to progress, which also have been found as contextual moderators (Smit et al. 2020). Peer support initiatives, while valuable, may need refinement to address the specific needs of individuals during societal recovery. Any type of support should include an empathetic environment where attention is paid to the individual's strengths, needs, wishes and agency while offering concrete, realistic, and practical perspective while facilitating in‐depth self‐discovery and offering hope. Some urge for a perspective shift in (mental)healthcare from the nano and micro‐context (the individual) towards a broader macro‐context (Furst et al. 2021). This perspective suits the recovery process as it also moves from the intra‐individual level (personal recovery) towards the inter‐individual level and societal context (societal recovery). How the recovery process specifically develops across these levels will be an interesting area of future research.

### 5.6 Strengths and Limitations

The strengths of this qualitative study lie in its ability to capture the depth and complexity of individual experiences. To limit bias, data was independently coded by two coders until reaching consensus and results were discussed in monthly team meetings and verified through member‐checks. Even though we aimed for the inclusion of a heterogenous sample on multiple demographic (age, gender, education level, cultural background) and clinical characteristics (number of previous episodes), the final sample had a relatively high educational level, the majority identified as woman, and only two participants from non‐Dutch ethnicities were included, which might have affected results. The inclusion of two deviant cases (one with a single episode and one with a current depression) showed that recovery experiences were largely consistent across minor differences in clinical profiles. Furthermore, the findings of this qualitative study may be specific to the Dutch cultural and social context and experiences within the Dutch mental health care system (GGZ). Although sensitising concepts were used to guide the analysis, this may have constrained full inductive analysis by subtly shaping how codes were clustered and interpreted. As recovery remains an individual process, the results of this qualitative evidence remain explorative.

## 6 Conclusion

In conclusion, our study underscores the interrelatedness of personal recovery and societal recovery. It suggests that personal recovery in depression is an intrapersonal process of self‐discovery and self‐management, while societal recovery focuses on belonging through meaningful activities. Discovering values that are intrinsic to the ‘self’ appear essential in this process. Participants often experienced a transitional phase of societal participation, where low‐demanding tasks are prioritised over alignment with personal values to increase one's taxability and self‐confidence. During personal recovery an emphasis was placed on empathetic support transitioning towards more pragmatic support during societal recovery.

## 7 Relevance to Clinical Practice

These findings highlight the role of mental health professionals in supporting the personal and societal recovery process in depression. During personal recovery, an empathetic environment that fosters self‐insight, autonomy, and hope is particularly beneficial. In this phase, peer support opportunities can also be considered to offer additional support. In the phase of societal recovery, individuals require more pragmatic and concrete guidance to support meaningful participation. Professionals should also recognise that flexible, low‐demand activities may serve as a necessary intermediate step to rebuild confidence.

## Author Contributions

A.G. performed the interviews, coding, analyses and drafted the initial manuscript. D.S., J.V. and J.S. supervised the overall process of data collection and analysis and provided input and feedback on the manuscript. All authors read and approved the final manuscript as submitted.

## Funding

The study is funded by ZonMw (06360332110007), a Dutch organisation for financing health research and innovation. The funder is not involved in the design of the study and collection, analyses and interpretation of data and in writing the manuscript.

## Ethics Statement

The Commission for human related research [Commissie Mensgebonden Onderzoek Arnhem‐Nijmegen] waived ethical approval as the study [file number: 13679.28] was not deemed subject to the WMO law [Dutch; Wet Mensgebonden Onderzoek]. Consequently, ethical approval was obtained by the Internal Review Board (IRB), the Pro Persona Research Ethics Committee, which adheres to the Declaration of Helsinki.

## Consent

Written informed consent was obtained for publication from participants.

## Conflicts of Interest

The authors declare no conflicts of interest.

## Supporting information


**Appendix S1:** Interview guide.
Description of data: Semi‐structured interview guide on personal and societal recovery in depression.

**Appendix S2:** COREQ Checklist.
Description of data: COREQ (COnsolidated criteria for REporting Qualitative research) Checklist.

## Data Availability

The datasets generated and/or analysed during the current study are not publicly available due to protection of privacy of participants but are available from the corresponding author on reasonable request at Pro Persona Research. All our study‐related information is stored in secure folders with limited access. Electronic data files are stored on a file system with access restricted to designated researchers and data monitors.
