# Supplementary material for: Personal and Societal Recovery in Depression—A Qualitative Study
Source: Int J Ment Health Nurs. 2026 Mar 19;35(2):e70247. doi: 10.1111/inm.70247 (PMC13003010; doi:10.1111/inm.70247)
Supplement: Supplementary file 1 — Appendix S1: Interview guide. Description of data: Semi‐structured interview guide on personal and societal recovery in depression. [file INM-35-0-s001.docx]

**Additional file 1**

**Interview Guide for the Research Article:**

" Personal And Societal Recovery in People with Depression - A Qualitative Study "

| **Introduction** | Introduce yourself (the researcher)  Explain   - Introduction of the topic - The rationale and objectives of the study (and research project) - the interview at any time - Procedure of the interview - Expected duration: 1 hour - Anonymity, confidentiality, voluntary participation; possibility to ask for a break or end the interview without explanation - Informed consent |
| --- | --- |
| **Before starting the recording** | - Ask if everything is clear - Ask if there are questions - Notifying participant of start of the audio recording |
| **Start of the interview** | |
| **Reason for participation** | - How did you become aware of this study? - Can you tell me something about why you want to participate in this study? |
| **General experience with depression** | - Can you tell me something about your experience with depression? - How are you at this moment (refer to MINI if necessary)? |
| **Introduction of the topic of recovery** | *‘In this interview, we would like to focus on your experience with personal and social recovery. This is not so much about the reduction of symptoms, but the process of (re)discovering your own identity, giving meaning to the depression and your life story, perspective, hope and connectedness with others. It may also involve your experience with the impact of depression and work, study, and social roles and contact.’* |
| **Process of recovery in general** | - Can you share what recovery means to you? - What are your experiences with recovery? - Can you describe the course of your recovery, what does this path look like for you? - How has the recovery process developed over time? - What are the signs for you that you are on the right path in the recovery process? - What have you learned through your recovery process? - How did this learning process come about? - And what has helped in that change? - What else would be needed in this process of recovery? - What has hindered or impeded your recovery process? - What have you missed in the recovery process? |
| **Societal recovery** | - Can you tell me about your daily activities? - What gives you satisfaction in a day? - What is your experience in the areas of work, housing, study, daily activities, and social contacts? (do not mention them all at once) - How has depression affected your daily activities? - How has depression affected your work or studies? - How has depression affected your social contacts? - How do you approach structuring daily activities when recovering from depression? - What is the importance of (meaningful) daily activities in the recovery process for you? - What has helped you in this regard? Or what do you think would be helpful? - What has hindered you or what do you perceive as possible obstacles? |
| **Personal vs. Societal recovery** | - Recovery is often referred to as personal and social recovery. Can you explain what the difference is for you? - Do you think these are separate processes? - How are these processes intertwined? - How are personal and social recovery connected in your recovery process? |
| **Recovery support – peers/experts by experience/close contacts** | - What support have you had in this process from peers, loved ones, and/or experts by experience? - How have you experienced this support? - What was helpful in this support? - What was hindering in this support? - In what way did these (not) contribute to your recovery? - What would further help you in this process towards a meaningful daily life? - What did you miss in this process? |
| **Recovery support – recovery initiatives** | - Have you participated in any specific (recovery) initiatives in your recovery process? - Did you make use of any particular methods? - How have you experienced this support? - How has this contributed to your recovery? - What was helpful in this support? - What was hindering in this support? - In what way did these (not) contribute to your recovery? - What would further help you in this process towards a meaningful daily life? - What did you miss in this process? |
| **Role of professional support (mental healthcare) in recovery** | - Have you been in treatment for mental health problems during the recovery process? - In what way has this (or not) contributed to your recovery process? - What was helpful in this treatment? - What was hindering in this treatment? - What would further help you in treatment in this process towards a meaningful daily life? - What have you missed in this treatment? |
| *Conclusion of interview* | |
| **Concluding the interview** | - Do you have anything to add to this conversation? - Did we miss something, topics that we did not discuss, but are important to you in recovery from depression? - How did you experience this interview? |
| *‘Thank you for participating in this study, and for openly sharing your experience. I will end the recording now’.* | |
| **Final comments** | - Invitation member-check - Future research participation - Questions or remarks afterwards - Contact details |
